# The Effect of Amoxicillin with Clavulanate on the Microbiota of Tonsillar Tissue in Disease: a Randomized Control Trial

**DOI:** 10.1128/spectrum.01239-22

**Published:** 2022-11-01

**Authors:** James Johnston, Brett Wagner Mackenzie, Kristi Biswas, Sharon Waldvogel-Thurlow, Sita Tarini Clark, Fiona Radcliff, Murali Mahadevan, Richard G. Douglas

**Affiliations:** a Department of Surgery, University of Auckland, Auckland, New Zealand; b Department of Molecular Medicine and Pathology, University of Auckland, Auckland, New Zealand; University of Michigan-Ann Arbor

**Keywords:** tonsil, tonsillectomy, microbiota, antibiotics, amoxicillin with clavulanate, recurrent tonsillitis, pediatrics

## Abstract

Despite antibiotics being the primary medical treatment for recurrent tonsillitis, the impact of antibiotics on the tonsillar microbiome is not well understood. This study aimed to determine the effect of amoxicillin with clavulanate on the composition and quantity of bacteria in the tonsils of children with recurrent tonsillitis. A multicenter randomized clinical trial in Auckland, New Zealand was undertaken between August 1, 2017, and June 30, 2018. Sixty children undergoing tonsillectomy for the indication of recurrent tonsillitis were recruited for this study. Following random allocation, 30 participants were prescribed amoxicillin with clavulanate for the week before surgery. The remaining 30 received no antibiotics. Immediately following surgery, the crypts of the right and left tonsils were swabbed. Bacterial 16S rRNA gene-targeted amplicon sequencing and histological techniques were utilized. In the control group, there were significantly higher relative abundances of Haemophilus, Streptococcus, *Neisseria*, and *Porphyromonas*. Members from the genera *Fusobacterium* and Treponema were found to be significantly more abundant in the antibiotic group. There were no significant differences in the absolute quantities of bacteria between the groups. Microscopic examination found fewer bacterial microcolonies present in the tonsillar crypts of participants in the antibiotic group. Streptococcus pyogenes was not present in these bacterial microcolonies. These results suggest that a single course of antibiotics has a significant impact on the tonsil microbiota composition. The duration of this effect and the effect that the altered microbiome has on the course of the condition need to be determined.

**IMPORTANCE** Several studies have identified the presence of multiple pathogenic bacteria in hyperplastic adenoids and palatine tonsils. However, there are currently no studies that utilize this technology to investigate the effect of oral antibiotics in children with recurrent tonsillitis on the tonsillar microbiome. This is the first study to investigate the effect of antibiotics on the microbiome of tonsillar tissue in children with recurrent tonsillitis using molecular techniques. This study has shown that participants who received amoxicillin with clavulanate immediately before tonsillectomy had a significantly reduced number of bacterial taxa commonly associated with recurrent tonsillitis, as well as the number of bacterial microcolonies observed in the tonsillar crypts. This novel finding suggests that either the effect of antibiotics is not sustained or that they are not an effective treatment for recurrent tonsillitis.

## INTRODUCTION

Recurrent tonsillitis (RT) occurs when repeated episodes of acute tonsillitis are interrupted by intervals of no or insignificant symptoms (1). Currently, the diagnosis of RT is based on clinical scoring systems, including the Centor score and McIsaac score, that determine the probability of a patient having group A streptococci (GAS) (1–4). Children who present with a sore throat and a McIsaac score of ≥3 are recommended to receive oral antibiotic therapy, most commonly penicillin V or phenoxymethylpenicillin-benzathine (1, 5). Advantages of the use of antibiotics in RT include a decreased duration of contagiousness, faster symptom resolution, reduction in purulent complications, and potential avoidance of immunogenic secondary diseases, including acute poststreptococcal glomerulonephritis and acute rheumatic fever (1, 6). Disadvantages of antibiotic usage include the significant problem of antibiotic overprescription for pharyngitis and tonsillitis and associated antimicrobial resistance.

Current guidelines recommend that antibiotic therapy is indicated only in cases of proven or highly suspected GAS infection. Microbiological diagnostic tests, including rapid antigen detection tests, microbial culture, multiplex PCR, and the streptococci antibody test (1) are often unavailable, expensive, or overly time consuming to be feasible in the community setting. Several studies have observed in practice that guidelines are not strictly adhered to and that antibiotics tend to be overused (7–9). This occurs despite the global problem of increasing antibiotic resistance (10).

Knowledge of the tonsillar microbiome has been enhanced by the utilization of bacterial 16S rRNA gene-targeted amplicon sequencing (11–13). To date, seven culture-independent molecular surveys based on 16S rRNA gene pyrosequencing have been completed to determine the microbiota on the surface and within the tissue of adenoids and palatine tonsils (11–18). These studies have identified the presence of multiple pathogenic bacteria in hyperplastic adenoids and palatine tonsils. In this study, we used bacterial 16S rRNA gene amplicon sequencing, Droplet Digital PCR (ddPCR), and histology to investigate the differences in tonsil microbiota composition, bacterial load, and presence of microcolonies in children with RT following a course of amoxicillin with clavulanate taken immediately before tonsillectomy.

Amoxicillin with clavulanate is not recommended as first-line therapy for the treatment of RT (1, 19, 20). This antibiotic was selected for this study given that there is evidence to suggest that amoxicillin with clavulanate is superior to penicillin both at eradicating *Streptococci* and non-*Streptococci* in RT (21). This is relevant given that GAS is only involved in a minority of complicated RT cases (19, 22). Also, it remains unclear which bacterial pathogens are associated with the majority of RT cases (20). A recent systematic review recommended the use of amoxicillin with clavulanate as an appropriate intervention in designing a randomized control trial such as this (20). This was advised given its broad bacterial spectrum, well-known side effects, cost, and oral route of administration (20). These recommendations were carefully considered before the selection of amoxicillin with clavulanate for this study.

## RESULTS

### Participant information.

**FIG 1:**
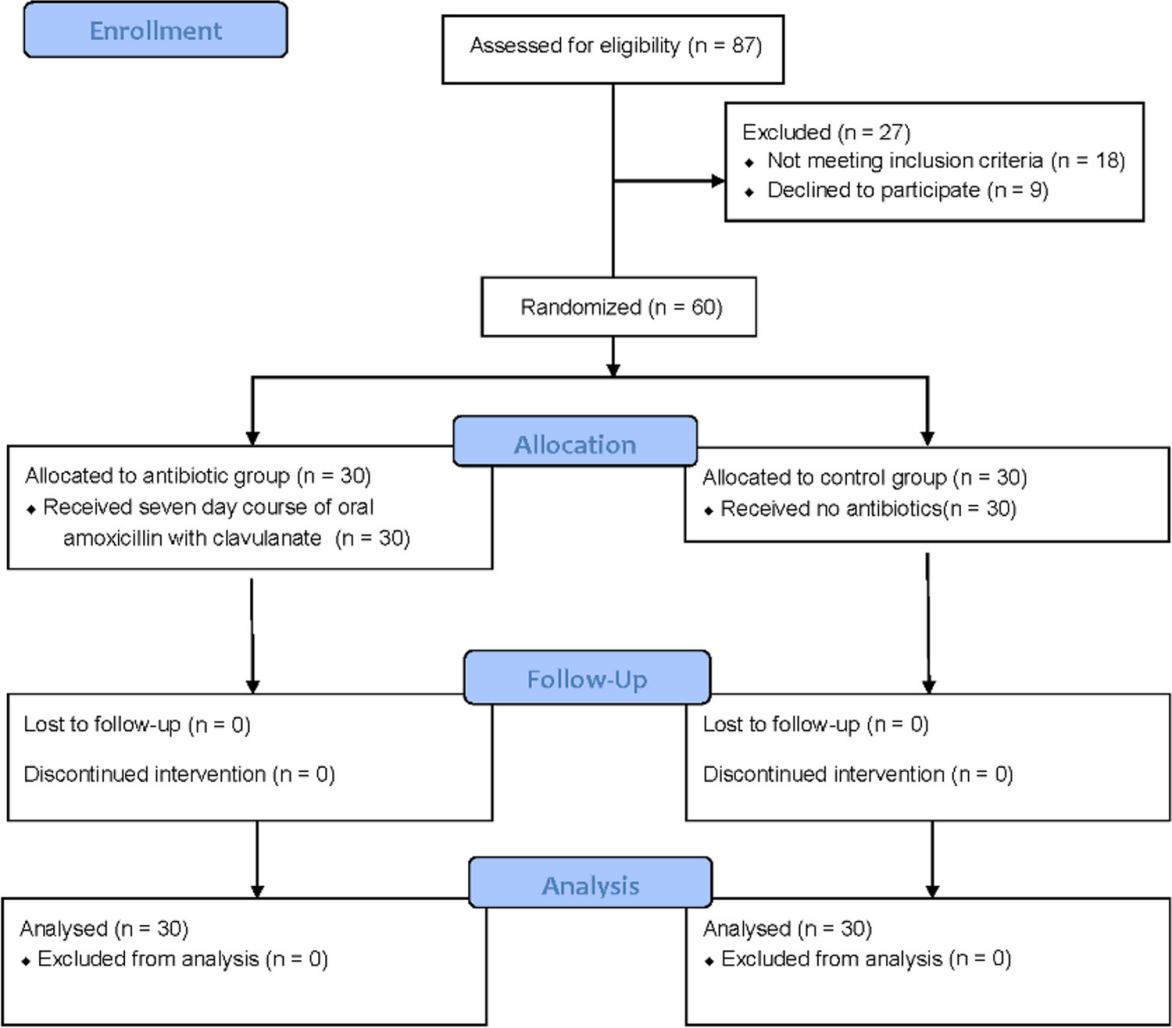
Flowchart of study population.

A total of 87 participants were registered, of whom 60 were randomized, and 60 (100% [30 in the antibiotic group and 30 in the control group]) completed the study (Fig. 1). The demographics and clinical characteristics of the 60 participants are shown in Table 1. Medications prescribed in the year preceding surgery for each participant were obtained by accessing community prescribing data, which are available to clinicians nationwide in New Zealand. No significant differences were noted in the number of courses of antibiotics prescribed between groups in the year before surgery (mean antibiotic intake, 3.7 ± 0.4; control, 3.6 ± 0.5). This was also the case for the prescription of analgesics and prednisone.

**TABLE 1:** Demographic and clinical characteristics of participants in the antibiotic group versus those in the control group

Characteristic	Antibiotic (*n* = 30)	Control (*n* = 30)	*P* value
Gender			
Female	60%	63%	*P *= 0.79
Mean age (yr)	8.7 ± 1.3	8.8 ± 1.4	*P *= 0.97
Mean body mass index	24 ± 2.1	23 ± 1.9	*P *= 0.55
Tonsil grade (Brodsky grading system)	2.8 ± 0.2	2.7 ± 0.2	*P *= 0.64
Asthma	13%	17%	*P *= 0.72
Eczema	50%	57%	*P *= 0.61
Allergic rhinitis	20%	27%	*P *= 0.54
Courses of antibiotics^*a*^	3.7 ± 0.4	3.6 ± 0.5	*P *= 0.67
At least one course of prednisone before surgery	3%	7%	*P *= 0.55
Ibuprofen prescribed at the time of surgery	83%	97%	*P *= 0.08
Tramadol prescribed at the time of surgery	73%	87%	*P *= 0.19

a Total number of courses of antibiotics prescribed before this study.

A general practitioner performed a throat swab for the detection of GAS in 97% of participants in the antibiotic group and 87% in the control group in the year preceding surgery. These swab results were positive for GAS in 60% of participants in the antibiotic group and 57% in the control group in the year before surgery. A complication was defined as a related admission to the hospital ≤30 days of discharge. There was no difference in 30-day readmission rates between the two groups.

### Bacterial community composition and diversity.

**FIG 2:**
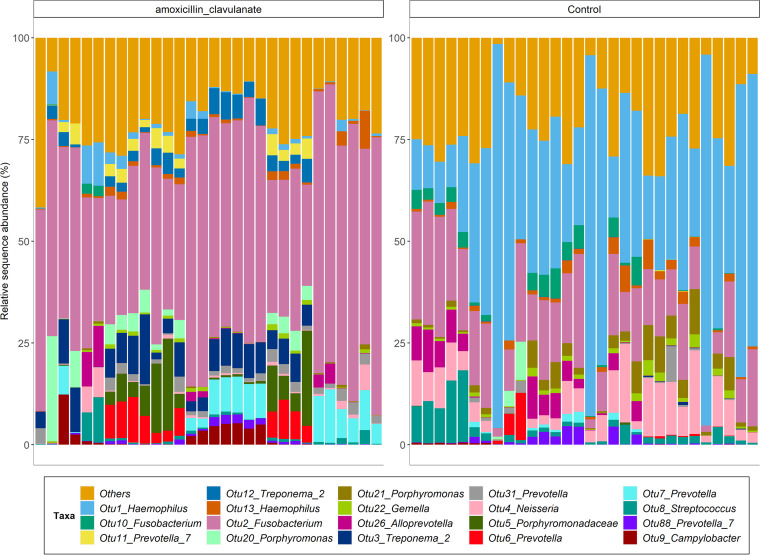
Relative sequence abundances of the 19 most abundant taxon-assigned operational taxonomic units (OTUs) from bacterial 16S rRNA gene amplicon sequencing recovered from tonsillar crypt swab amplicon sequencing data in all patients from this study (antibiotic = 30; control = 30). All other taxon-assigned OTUs are grouped in “Others.”

Tonsil crypt swabs were used to compare the antibiotic group with the control group. In the antibiotic group at the phylum level, the tonsil swabs were dominated by *Fusobacteria*, *Spirochaetes* (Treponema), and *Bacteroidetes* (*Prevotella* and *Porphyromonas*). In the control group at phylum level, the tonsil swabs were dominated by *Proteobacteria* (Haemophilus and *Neisseria*), *Fusobacteria* (*Fusobacterium*), *Firmicutes* (Streptococcus), and *Bacteroidetes* (*Prevotella* and *Porphyromonas*). Considerable variability in bacterial community composition between groups was observed at the operational taxonomic unit (OTU) level (Fig. 2).

**FIG 3:**
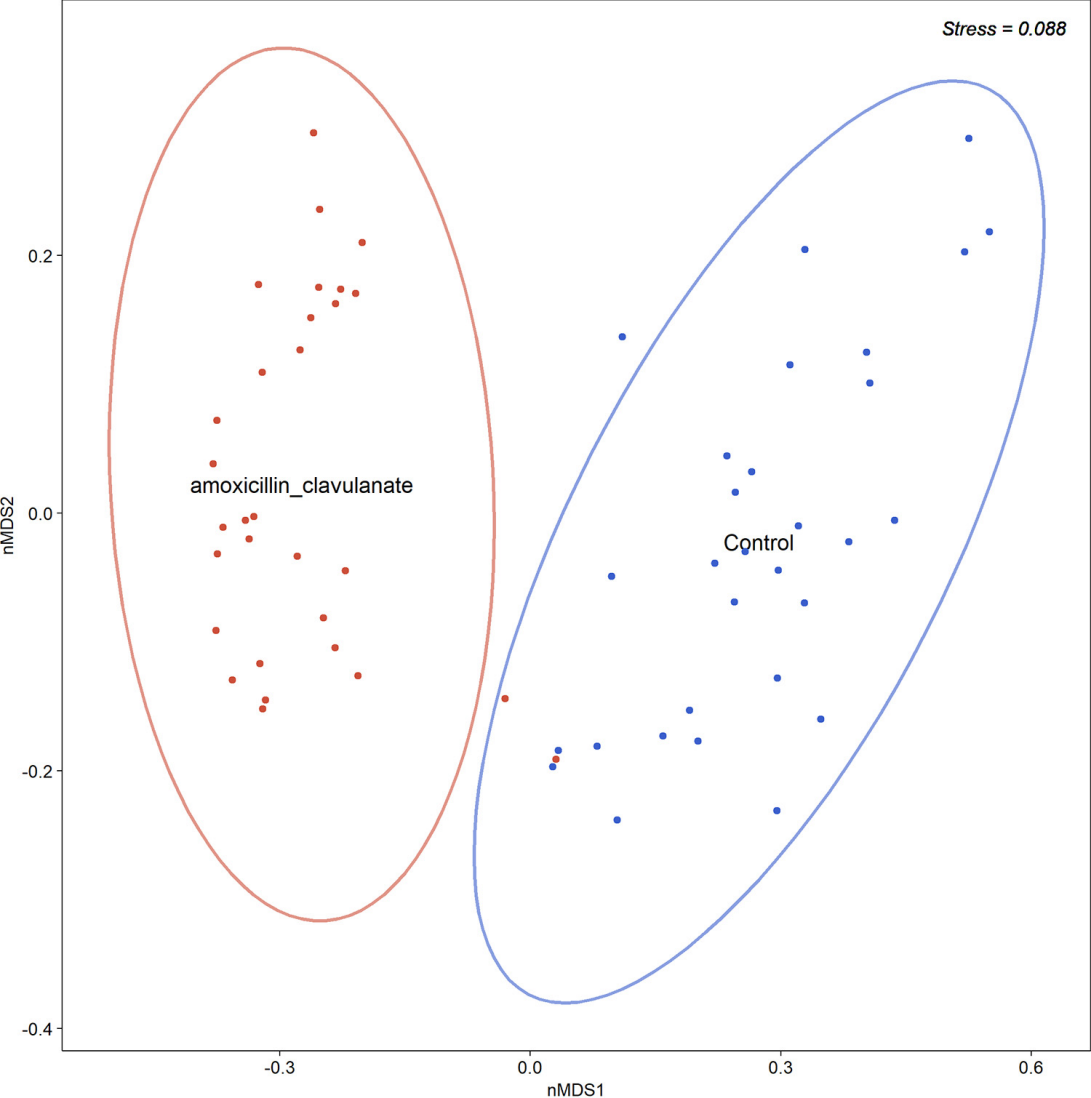
Bray-Curtis dissimilarity nonmetric multidimensional scaling (nMDS) plot of antibiotic-treated (*n* = 30) and control (*n* = 30) samples. Ellipses represent the mean of the description coordinates at the center, and the dispersion of the ellipses was calculated using the standard deviation of the weighted average of covariance matrix group scores. The multivariate homogeneity of group dispersions with Tukey’s honestly significant difference did not indicate a significant difference of dispersion between groups, *P > *0.05.

Shannon, Jost1, and Simpson alpha diversity indices that account for both abundance and evenness were not significantly different between the microbial communities of the two groups (*P* > 0.05 for all). However, the species richness of tonsil crypts in the control group were significantly more diverse compared to those from the antibiotic group (Wilcoxon rank sum test, *P* = 0.001). Distinct clustering of samples by cohorts was observed in the nonmetric multidimensional scaling plot (Fig. 3). No significant difference in the dispersion of samples within groups was observed.

**FIG 4:**
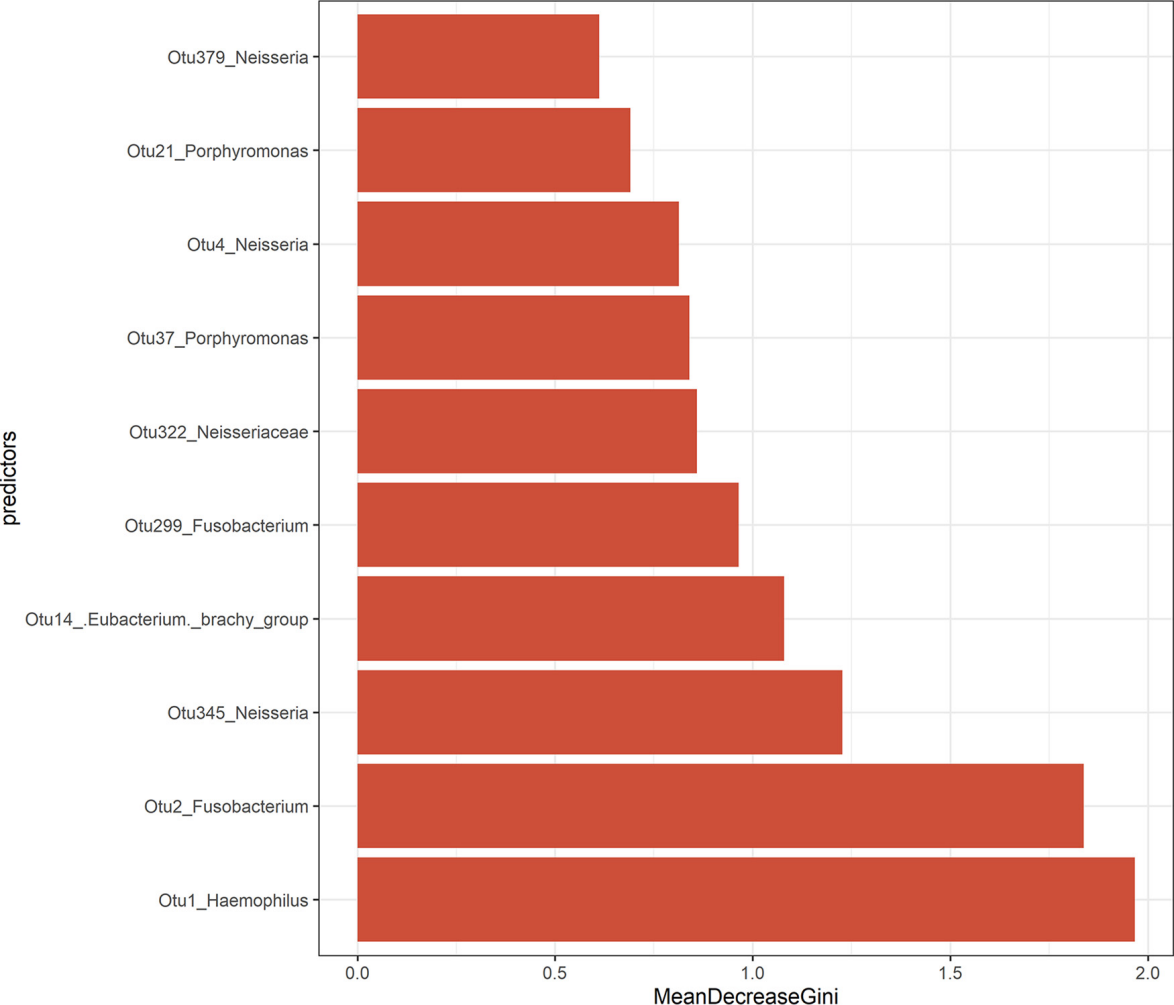
Random Forest unsupervised testing: most important variables for classifying tonsillar crypt swab samples according to antibiotic usage. The top 10 most important taxon-assigned OTUs from the Random Forest classification model are organized according to the decrease in the Gini coefficient.

Permutational analysis of variance suggested that antibiotic treatment contributed a large, significant proportion to the global differences in bacterial community structure (*R*^2^ = 44.8%, *P* = 0.001). Pairwise comparisons between groups for the top 100 OTUs were analyzed using Dunn’s test of multiple comparisons with Bonferroni adjustment to assess which OTUs could be driving the global differences. This revealed higher relative abundances of Streptococcus (*P* = 0.002), Haemophilus (*P* < 0.001), *Neisseria* (*P* < 0.001), and *Porphyromonas* (*P* < 0.001) in the control group. In contrast, higher relative abundances of *Fusobacterium* (*P* < 0.001), *Prevotella* (*P* < 0.001), Treponema (*P* < 0.001), and *Parvimonas* (*P* < 0.001) were detected in the antibiotic group. Random Forest unsupervised testing was applied to evaluate classification of samples by OTUs according to whether or not the patient was prescribed antibiotics. In this model, the classification accuracy was 95% with an “out-of-bag” estimate of an error rate of 5% (29/30 samples from the control group were classified correctly compared to 28/30 in the antibiotic treatment group). The most important OTU predictors were OTU1 Haemophilus and OTU2 *Fusobacterium* (Fig. 4). These data support those from Dunn’s tests.

**FIG 5:**
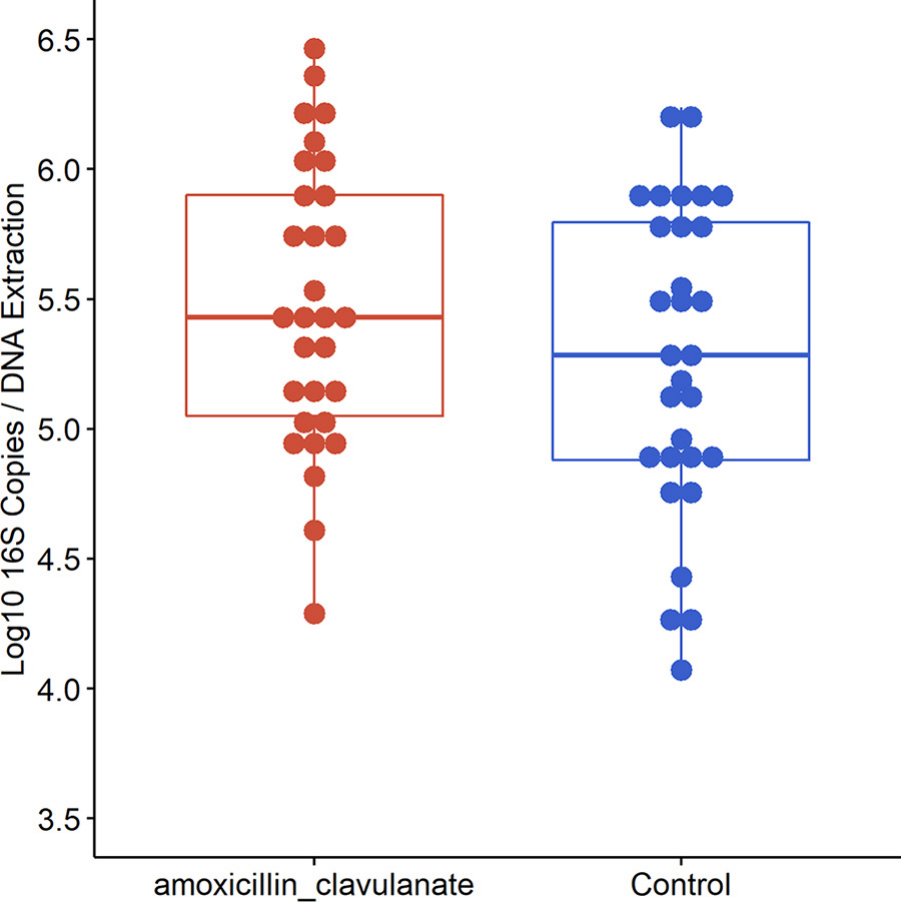
Box plots showing comparisons of log_10_-transformed bacterial loads between antibiotic-treated and control patients measured using Droplet Digital PCR. Median values are indicated by the solid line within each box, and the box extends to upper and lower quartile values; outlier data points are indicated by closed circles. Comparisons between groups were assessed using Welch’s two-sample *t* test, and no significant difference was detected (*P > *0.05).

ddPCR analyses revealed no significant difference in the number of 16S rRNA gene copies between groups (Welch two-sample *t* test, *P* > 0.05) (Fig. 5).

### Histological analyses.

**FIG 6:**
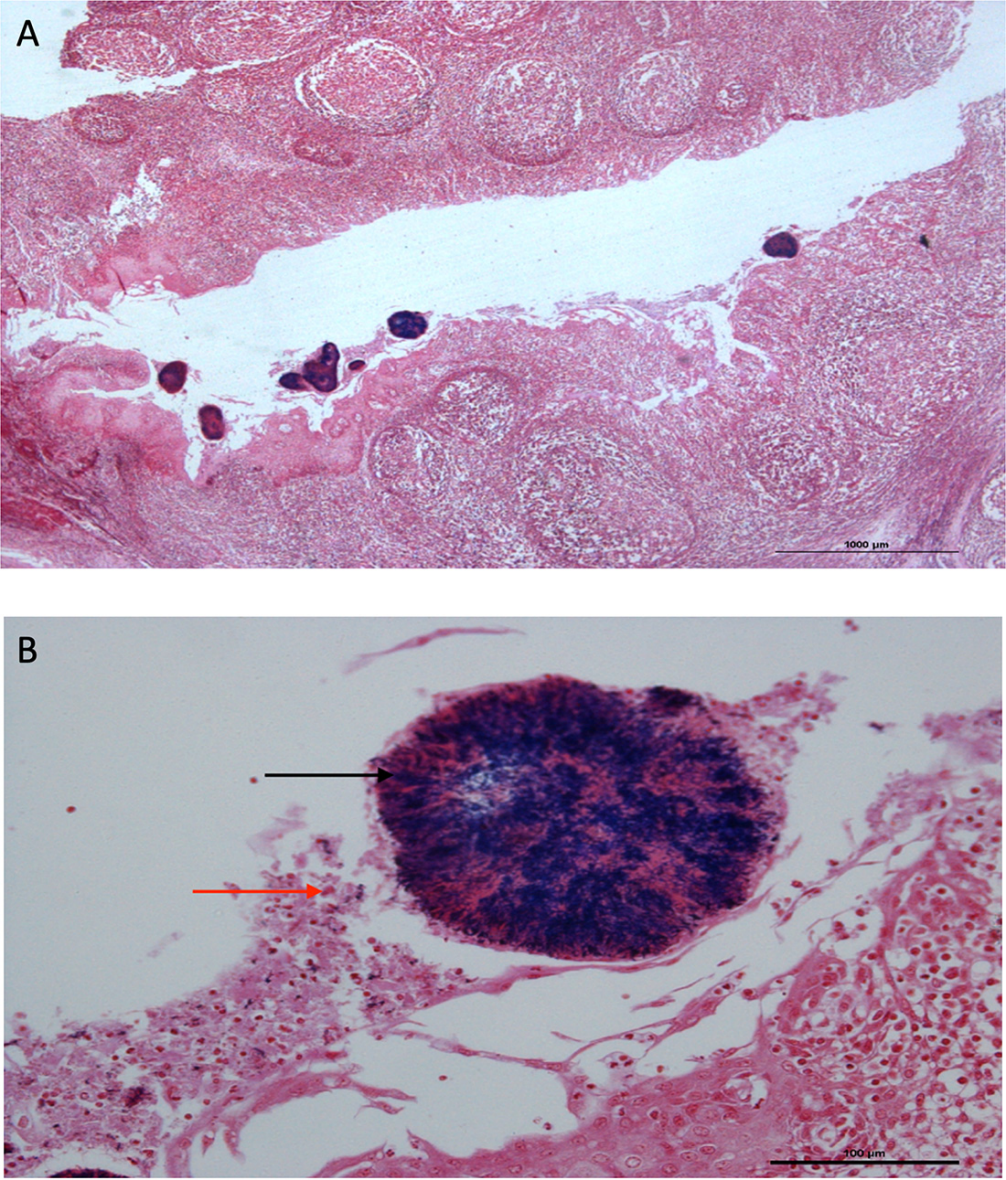
(A) Demonstration of bacterial microcolonies surrounded by B and T lymphocytes in the tonsil crypt of a patient in the control group at ×40 magnification. (B) Demonstration of the same bacterial microcolony surrounded by B and T lymphocytes in the tonsil crypt of a patient in the control group at ×60 magnification. Black arrow denotes bacteria in a microcolony. Red arrow denotes a group of lymphocytes.

All available histological slides were reviewed for the presence of bacterial microcolonies (Fig. 6A and B). There were significantly more Gram-positive bacterial microcolonies observed in the control group (mean number = 60 ± 48.8; range, 13 to 144) compared with the treatment group (mean number = 3.6 ± 3.2; range, 0 to 10) (*P* = 0.02). There was no significant difference in the number of Gram-negative microcolonies observed in the control group (mean number = 1.7 ± 3.1; range, 0 to 9) compared to the antibiotic group (mean number = 0.3 ± 0.5; range, 0 to 1) (*P = *0.29). Bacterial microcolonies were reviewed for the presence of S. pyogenes using immunohistochemistry. S. pyogenes was not observed in any of the microcolonies in any of the participant's tonsil samples.

## DISCUSSION

To our knowledge, this is the first study to investigate the effect of antibiotics on the microbiome of tonsillar tissue in children with RT using molecular techniques. We observed a significant difference in the composition of the microbiota of tonsils from children who received a 1-week course of amoxicillin with clavulanate compared with those who did not. The bacterial communities in the antibiotic group were dominated by *Fusobacterium* and Treponema, compared with tonsils from the control group, which were dominated by Streptococcus, Haemophilus, *Neisseria*, and *Porphyromonas*. Amoxicillin with clavulanate significantly reduced the abundance of several bacterial taxa commonly associated with RT and the overall bacterial richness. Interestingly, there was no significant difference between the two groups in the number of bacterial 16S rRNA gene copies recovered from swab samples. We hypothesize that opportunistic bacterial genera replaced bacteria that were eradicated by amoxicillin with clavulanate.

In this cohort, we observed a substantial reduction in the number of bacterial microcolonies in the tonsillar crypts of the patients who were prescribed amoxicillin with clavulanate. We did not observe any S. pyogenes within the microcolonies of participants in this cohort, although the genus Streptococcus was detected in the sequencing data. This was a very unexpected finding, as RT is widely accepted as a disease primarily caused by *Streptococcal* infection (4). It is likely that the limited amount of Streptococcus seen in our participants is related to the absence of infective signs and symptoms and the time of sampling (tonsillectomy). This may imply that the tonsils are not chronically colonized with Streptococcus in between infective episodes.

The results from this study show that the effects of one course of amoxicillin with clavulanate are observed within 1 week. However, children with RT are prescribed several courses of antibiotics and ultimately require a tonsillectomy. The clinical implications are that either the effect of treatment is not sustained or the type and dosages prescribed do not effectively impact the progression of disease.

The cross-sectional design of this study limits the conclusions that can be drawn from the results. Future studies should examine the effects of antibiotic treatment on the longitudinal stability of tonsillar bacterial community composition. Another limitation of RT research is the paucity of samples from healthy controls with no history of RT or tonsillar hyperplasia. The significance of bacterial microcolonies in the tonsillar crypts in the context of disease is merely speculation without comparisons to healthy control samples. Future research should focus on the effects of other antibacterial agents, the duration of treatment, the duration of the effect of antibacterial treatment, and the accumulation of antibacterial resistance genes as a result of several courses of antibiotics for the treatment of RT.

### Conclusions.

We have demonstrated a significant alteration in the bacterial communities of tonsil tissue following a course of amoxicillin with clavulanate in children with RT. Amoxicillin with clavulanate significantly reduced bacterial taxa commonly associated with RT and the number of bacterial microcolonies observed in the tonsillar crypts. Despite these significant alterations in the microbiota, these patients still required a tonsillectomy. This suggests that either the effect of antibiotics is not sustained or that they are not an effective treatment for RT.

## MATERIALS AND METHODS

### Study design, participants, and intervention.

This randomized, nonblinded clinical trial of the administration of amoxicillin with clavulanate to children with RT was conducted between August 1, 2017, and June 30, 2018, in two centers in Auckland, New Zealand. These two centers are major public hospitals that provide tertiary-level pediatric otolaryngology services in the region.

Sixty children undergoing tonsillectomy for the indication of RT were recruited for this study. The necessity for tonsillectomy was based on the Paradise criteria (23) which defines clinically significant RT (requiring tonsillectomy) as seven episodes in 1 year, five events per year for 2 years, or three or more episodes per year for 3 years (20, 23). Participants ≤16 years and had taken no antibiotics for ≥8 weeks before the commencement of this study were included. All participants have previously had at least one lifetime dose of amoxicillin with clavulanate to ensure the risk of adverse reaction was minimized. Thirty participants were randomly allocated to the antibiotic group and prescribed a 7-day course of amoxicillin with clavulanate immediately before and including the day of surgery. The remaining 30 participants were randomly assigned to the control group and received no antibiotics. Compliance in the treatment group was self-reported by participants. Additionally, a community prescribing database was checked to ensure they had collected their prescription from the pharmacy and a phone call was made midway through the week to ensure everything was going well with taking the medication. Written consent was obtained from the legal guardian of each participant, and ethical approval from the New Zealand Health and Disability Ethics Committee (17/NTB/76) was received for this study. The trial was registered with the Australian New Zealand Clinical Trials Registry (ANZCTR) and was reported using CONSORT guidelines. The ANZCTR number is ACTRN12618001847202 and was retrospectively registered.

Recorded demographic data included age, sex, body mass index, and ethnicity. Clinical data included the tonsil grades (2), medical comorbidities, antibiotics prescribed in the community in the year before surgery, and GAS results from throat swabs for culture before admission. Outcome data included postoperative visits to the general practitioner, 30-day readmission rate, and associated complications.

### Randomization and blinding.

During the recruitment phase, children were randomized in a 1:1 ratio using a computer-based randomization system (randomizer.org). This was an open-label study; study investigators and legal guardians knew which group they were allocated to. No placebo was used.

### Sample collection.

Tonsillectomy was performed under general anesthetic by a pediatric oral surgeon. No patients were experiencing symptoms or clinical signs of tonsillitis at the time of surgery. Intravenous antibiotics were not administered at induction. Following induction, extracapsular tonsillectomy was performed with either bipolar diathermy or coblation diathermy (Smith & Nephew, London, UK). Each tonsil was removed and placed immediately into individual sterile pots. These samples were stored on ice and taken to the laboratory for processing.

Preoperative tonsil swabs were not collected as it is not practical to obtain an adequate tonsil crypt swab in a child before tonsillectomy without discomfort and potential contamination. To accurately sample the palatine tonsils, swabs were taken deep within the tonsil crypts immediately following tonsillectomy. Pairs of sterile, rayon-tipped swabs (Copan; number 170KS01) were used to swab the crypts of each palatine tonsil and then placed in a single sterile 1.5-mL Eppendorf tube containing RNAlater. Pairs of swabs were stored in a single tube and processed together. All tubes were stored at −20°C until further analysis.

### Total DNA extraction and bacterial 16S rRNA gene sequencing.

Total DNA was extracted from the swabs, and the bacterial 16S rRNA gene was amplified and sequenced as previously described (13). Negative PCR controls with PCR-grade water were included for all PCRs, with no detectable PCR product. Equimolar concentrations of amplicon samples were submitted to Auckland Genomics Ltd., Auckland, New Zealand for library preparation and sequencing on the Illumina MiSeq platform (2 × 300-bp paired-end reads).

### Bioinformatic analyses.

Raw sequence reads were quality filtered, as described previously (11). Briefly, primer binding regions were trimmed, and sequences were merged and quality filtered in USEARCH v10 (24, 25). Sequences were clustered using the UCLUST algorithm into OTUs based on 97% sequence similarity (26). OTUs were taxonomically assigned using the LTP 16S SILVA database v 123 (27). Human-assigned reads were manually filtered from the data set and samples were normalized to 3,780 sequence counts per sample for further analysis. Alpha diversity metrics and Beta-diversity distance matrices were calculated in USEARCH. The permutational analysis of the variance “adonis2” function in R was used to analyze the impact of antibiotics on the bacterial community structure in tonsil samples (28).

### Droplet digital PCR.

Droplet Digital PCR (ddPCR) measures absolute quantities by counting nucleic acid molecules encapsulated in discrete volumetrically defined water-in-oil droplet partitions. An aliquot of extracted DNA from each sample was subjected to ddPCR to assess bacterial load indicated by the number of 16S rRNA gene copies, as previously described (29). Droplets were analyzed using the QuantaSoft software according to the manufacturer’s recommendations. Manual thresholds were set for droplet counts and the final counts were log_10_ transformed and plotted using the R statistical program.

### Histological analysis.

Following swab collection, the left palatine tonsils from a random subset of 14 participants (7 treatment and 7 control) were fixed in formalin and then embedded in paraffin wax. Each palatine tonsil was sectioned in the coronal plane at 250-μm intervals, with 4-μm thick sections cut at each point. A Gram stain was performed on each coronal section of the 14 tonsils, followed by a counterstain with safranin to identify Gram-negative bacteria. All sections were screened at ×40 magnification on a Leica DMR upright microscope looking for the presence or absence of bacterial microcolonies in each section. A bacterial microcolony was defined as a colony of bacteria visible only under a low-power microscope (×40 magnification).

Tonsil sections containing bacterial microcolonies underwent further histological analysis. Each adjacent coronal section was stained by immunofluorescence for the presence of S. pyogenes. Sections were blocked in 10% normal goat serum before overnight incubation of 1:5,000 dilution of Rabbit anti-Streptococcus A (Biorbyt, Cambridge, UK) antibody at 4°C. The following day the secondary antibody goat anti-rabbit 488 (Invitrogen, Carlsbad, CA, USA) was applied. Nuclei were stained with DAPI and sections were mounted in Citifluor. A tonsil containing S. pyogenes was used as a positive control with each batch of staining. All sections were screened for the presence of S. pyogenes on a Leica DMR microscope at ×64 magnification.

### Statistical analysis.

Participant demographics and clinical characteristics were summarized using descriptive statistics. Univariate analysis was used to assess potential factors associated with differences in participants based on allocation to antibiotic or control group. Chi-square tests were performed to evaluate categorical variables, and Student’s *t* test was conducted to evaluate continuous variables. A two-tailed *P* value of <0.05 was regarded as statistically significant. IBM SPSS version 24 software was used for all statistical analyses.

Significant differences between amoxicillin with clavulanate treated and control patients’ bacterial communities and total bacterial load were assessed using the R statistical package. Specifically, data were initially assessed for normality using the Shapiro-Wilks test, followed by a *t* test or Wilcoxon Rank Sum test for normal or nonnormally distributed data, respectively. The multivariate homogeneity of group dispersions was assessed for significant differences between groups with Tukey’s honest test. Differences in the relative abundance of OTUs were calculated using the Kruskal-Wallis test, followed by pairwise testing via Dunn’s test of multiple comparisons with Bonferroni adjustment. The randomForest unsupervised test was implemented in R using the randomForest package version 4.7-1.1. All OTUs were used in the model as predictor variables, and the antibiotic prescription was set as the response variable. A Random Forest classification with 10,000 trees was implemented with 21 variables tried at each split, and the model performance was estimated using an “out-of-bag” error. The top 10 predictor OTUs were plotted using ggplot2.

### Data availability.

The sequencing data are available through the NCBI SRA under accession number PRJNA591372.
